# The ferroptosis inducer erastin irreversibly inhibits system x_c_− and synergizes with cisplatin to increase cisplatin’s cytotoxicity in cancer cells

**DOI:** 10.1038/s41598-018-19213-4

**Published:** 2018-01-17

**Authors:** Mami Sato, Ryosuke Kusumi, Shinji Hamashima, Sho Kobayashi, Satoru Sasaki, Yuhei Komiyama, Takuji Izumikawa, Marcus Conrad, Shiro Bannai, Hideyo Sato

**Affiliations:** 1 Laboratory of Biochemistry and Molecular Biology, Department of Medical Technology, Faculty of Medicine, Niigata University, 746-2 Asahimachi-dori, Chuo-ku, Niigata 951-8518 Japan; 2 Department of Biochemistry and Molecular Biology, Graduate School of Medical Science, Yamagata University, Yamagata, 990-9585 Japan; 3 Department of Food and Applied Life Sciences, Faculty of Agriculture, Yamagata University, Tsuruoka, 997-8555 Japan; 4 Division of Radioisotope Research, CCRF, Institute for Research Promotion, Niigata University, 757-1 Asahimachi-dori, Chuo-ku, Niigata 951-8510 Japan; 5 Helmholtz Zentrum Muenchen, Institute of Developmental Genetics, 85764 Neuherberg, Germany

## Abstract

System x_c_^−^ was recently described as the most upstream node in a novel form of regulated necrotic cell death, called ferroptosis. In this context, the small molecule erastin was reported to target and inhibit system x_c_^−^, leading to cysteine starvation, glutathione depletion and consequently ferroptotic cell death. Although the inhibitory effect of erastin towards system x_c_^−^ is well-documented, nothing is known about its mechanism of action. Therefore, we sought to interrogate in more detail the underlying mechanism of erastin’s pro-ferroptotic effects. When comparing with some well-known inhibitors of system x_c_^−^, erastin was the most efficient inhibitor acting at low micromolar concentrations. Notably, only a very short exposure of cells with low erastin concentrations was sufficient to cause a strong and persistent inhibition of system x_c_^−^, causing glutathione depletion. These inhibitory effects towards system x_c_^−^ did not involve cysteine modifications of the transporter. More importantly, short exposure of tumor cells with erastin strongly potentiated the cytotoxic effects of cisplatin to efficiently eradicate tumor cells. Hence, our data suggests that only a very short pre-treatment of erastin suffices to synergize with cisplatin to efficiently induce cancer cell death, findings that might guide us in the design of novel cancer treatment paradigms.

## Introduction

System x_c_^−^ is one among many amino acid transporters expressed in the plasma membrane of mammalian cells^1^. This transporter is composed of xCT (SLC7A11), which is the substrate-specific subunit^2,3^, and 4F2 heavy chain (SLC3A2). xCT was shown to be responsible for the specific function of system x_c_^−^, whereas 4F2 heavy chain, which had been known as one of surface antigens (CD98), is the common subunit of some other amino acid transporters^4–6^. System x_c_^−^ exchanges intracellular glutamate with extracellular cystine at a 1:1 molar ratio^7^. Recently, we have demonstrated that cystathionine is also a physiological substrate, which can be exchanged with glutamate, and that system x_c_^−^ plays an essential role for maintaining cystathionine in immune tissues like thymus and spleen^8^. Cystine taken up via system x_c_^−^ is rapidly reduced to cysteine, which is used for synthesis of protein and glutathione (GSH)^9^, the major endogenous antioxidant in mammalian cells. Some part of cysteine is released via neutral amino acid transporters, thus contributing to maintain extracellular redox balance^10^, and a cystine/cysteine redox cycle which can act independently of cellular GSH^11,12^. Inhibition of system x_c_^−^ causes a rapid drop of intracellular glutathione level and cell death in most of cultured cells^13^.

Since the uptake of cystine and cystathionine is inevitably coupled to the release of glutamate, a major neurotransmitter in the central nervous system, system x_c_^−^ has been linked to a variety of normal functions and neurological diseases, such as Parkinson’s disease, Alzheimer’s disease, and amyotrophic lateral sclerosis^14^. In addition, system x_c_^−^ has recently emerged as a potential target in the context of cancer therapy^15^. In fact, many reports have demonstrated that inhibition or down-regulation of system x_c_^−^ function attenuates proliferation, invasion, and metastasis of cancer cells *in vitro* and *in vivo*^16^. Therefore, exploitation of specific and potent inhibitors of system x_c_^−^ is considered to be of potentially great benefit for cancer chemotherapy. In this regard, many compounds have been found as inhibitors of system x_c_^−^^17,18^. Among these, erastin (named for eradicator of RAS and ST-expressing cells) was first identified by synthetic lethal high-throughput screening by Stockwell’s group as a small molecule compound efficiently killing human tumor cells without affecting their isogenic normal cell counterparts^19^. Then, the same group discovered that erastin is a potent and selective inhibitor of system x_c_^−^ causing a novel iron-dependent form of non-apoptotic cell death, designated as ferroptosis^20,21^.

Yet, the mode of the inhibition of system x_c_^−^ by erastin has remained unclear. In the present study, we have investigated the inhibitory characteristics of erastin on the activity of system x_c_^−^ and intracellular glutathione levels, and found that erastin has a persistent inhibitory effect, which appears to be entirely different from other system x_c_^−^ inhibitors.

## Results

### Specificity of the inhibitory effects of erastin on system x_c_^−^ activity

**Figure 1:**
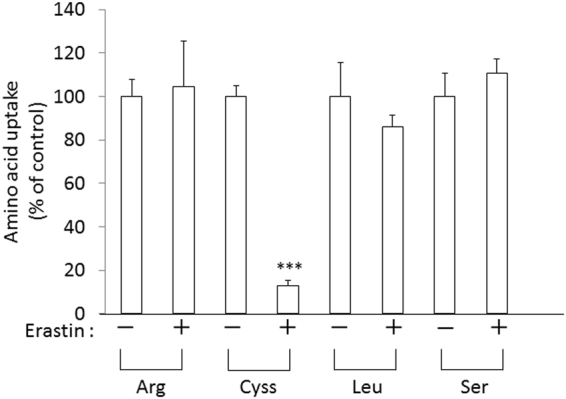
Effect of erastin on the activity of various amino acid transport systems in xCT-over-expressing MEF. xCT-overexpressing MEF were cultured for 24 h and then the uptake of 0.05 mM L-[^14^C]cystine (Cyss), L-[^14^C]arginine, L-[^14^C]serine, and L-[^14^C]leucine was measured in the presence of 10 µM erastin. Bars represent the mean of percentages ± S.D. (n = 5 for cystine uptake; n = 4 for uptake of other amino acids). P values were obtained by unpaired Student’s t test. ^***^P = 1 × 10^−6^.

To confirm that erastin specifically inhibits the activity of system x_c_^−^, we measured the activity of the uptake of arginine, leucine and serine in addition to cystine in the presence or absence of 10 μM erastin in xCT-overexpressing MEF (Fig. 1). No inhibition was detectable for arginine uptake (system y^+^), leucine uptake (system L), and serine uptake (system ASC), whereas cystine uptake was strongly impaired by erastin in xCT-overexpressing MEFs. These data unequivocally show that erastin selectively inhibits system x_c_^−^ and that it has no impact on other amino acid transport systems.

### Comparison of inhibitory efficiency of xCT inhibitors

**Figure 2:**
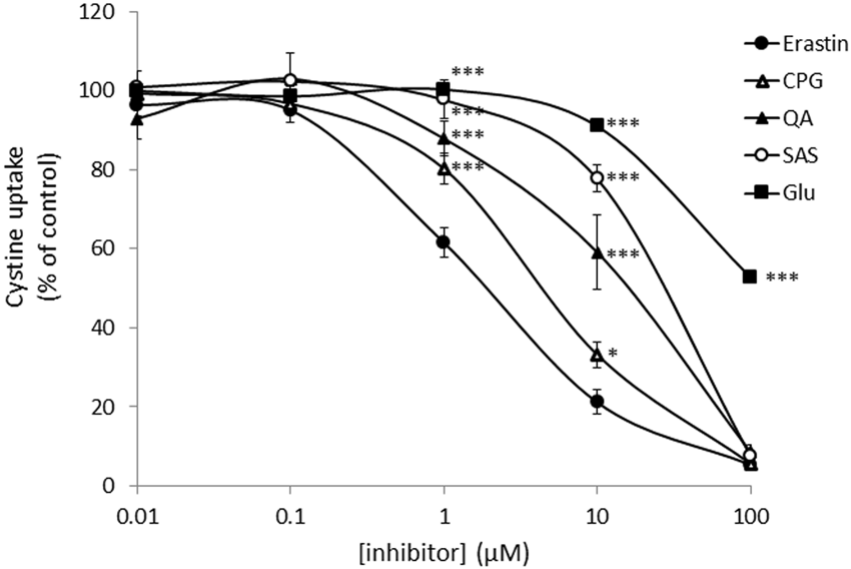
Effects of various xCT inhibitors on cystine uptake activity in xCT- overexpressing MEF. xCT-overexpressing MEF were cultured for 24 h and then the uptake of 0.05 mM L-[^14^C]cystine was measured in the presence of various inhibitors at the concentrations indicated. Each point represents the mean ± S.D. (n = 4 for CPG, QA, and Glu; n = 6 for Erastin and SAS). P values were obtained by one-way ANOVA followed by Tukey’s multiple comparison test. ^*^P = 1.8 × 10^−2^ (erastin vs CPG at 10 µM). ^***^P = 1 × 10^−6^ (erastin vs Glu or SAS at 1 µM, erastin vs Glu, SAS, or QA at 10 µM, erastin vs Glu at 100 µM), 6 × 10^−6^ (erastin vs QA at 1 µM), 4 × 10^−4^ (erastin vs CPG at 1 µM).

There are a number of small molecules inhibitors known that inhibit the uptake of cystine or glutamate into cells. We compared the inhibitory efficiency of these inhibitors in xCT-overexpressing MEF by determining the uptake of 0.05 mM cystine into cells. As shown in Fig. 2, sulfasalazine (SAS), (S)-4-carboxyphenylglycine (CPG), and quisqualic acid (QA) inhibited the cystine uptake as efficiently as erastin at the concentration of 100 μM. Yet, at lower concentrations erastin was by far more potent in inhibiting the uptake of cystine as compared to the other inhibitors tested. The calculated IC_50_ values of erastin, CPG, QA and SAS were 1.4 µM, 4.4 µM, 15.4 µM and 26.1 µM, respectively. L-glutamate is one of the physiological substrates of system x_c_^−^ and is known to competitively inhibit the uptake of cystine. At 100 µM glutamate only inhibited the cystine uptake by approximately 50%, indicating that the affinity of erastin towards xCT is much higher compared to physiological substrates.

### Comparison of persistent effects of xCT inhibitors

**Figure 3:**
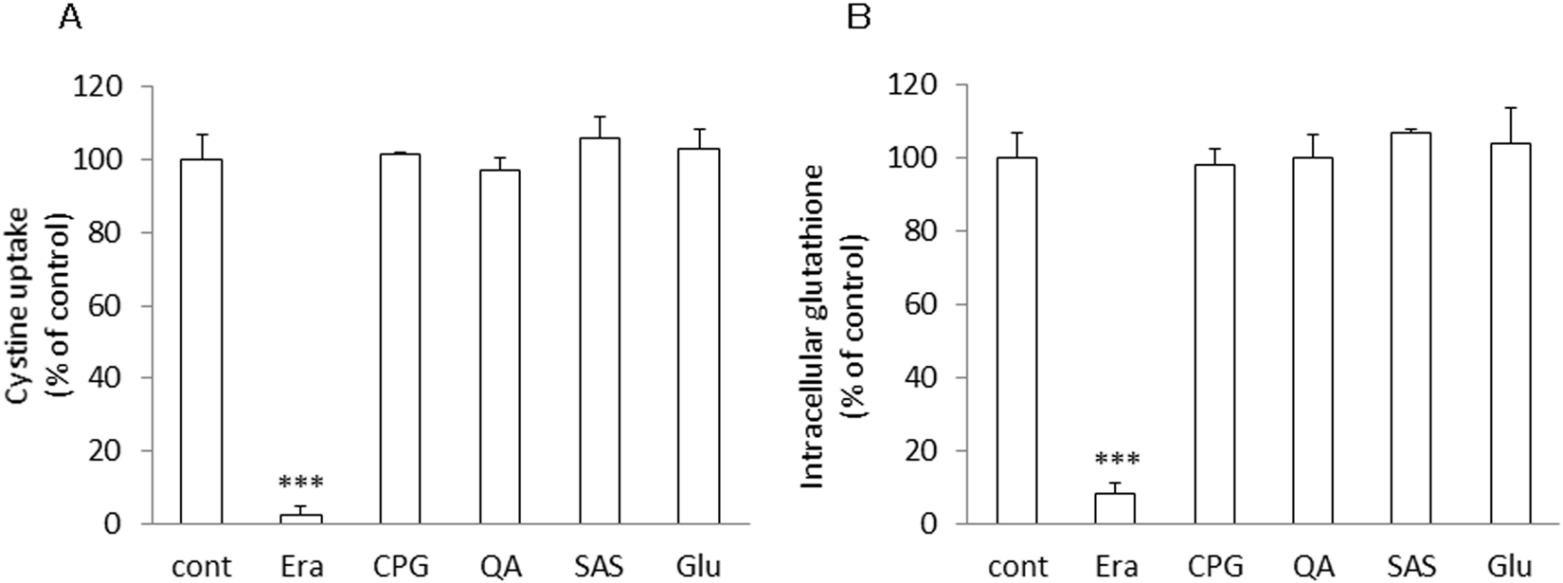
Persistent effects of various xCT inhibitors on the activity of cystine uptake and intracellular total glutathione in xCT-overexpressing MEF exposed for a short time. xCT-overexpressing MEF were cultured for 24 h and then exposed to the inhibitors at the concentration of 100 µM each for 5 min. After exposure, cells were washed with 1 mL of fresh medium once. 2 mL of fresh medium was added and cells were allowed to grow for another 24 h. Then, the activity of cystine uptake (**A**) and intracellular glutathione levels (**B**) were measured. Bars represent the mean of percentages ± S.D. (n = 8 for control; n = 4 for the inhibitors). P values were obtained by one-way ANOVA followed by Tukey’s multiple comparison test. ^***^P = 1 × 10^−6^.

At 100 µM most of the xCT inhibitors showed a similar inhibitory effect on the uptake of cystine. Therefore, we have further investigated the characteristics of the mode of inhibition of these reagents towards xCT. xCT-overexpressing MEF were exposed to 100 μM of each inhibitor for 5 min. After 5 min incubation, the cell culture medium was removed, cells were rinsed once with 1 ml of fresh medium and incubated with 2 ml of fresh medium lacking any inhibitors for 24 h. After 24 h, the cystine uptake was measured in the absence of the inhibitors. At the same time, intracellular glutathione levels were determined. As shown in Fig. 3A, the inhibitory effects of CPG, QA, SAS, and glutamate towards xCT were completely abolished 24 h after exposing cells for 5 min to these compounds, while in stark contrast the uptake of cystine was still inhibited dramatically in the cells which had been exposed for just 5 min with erastin. Accordingly, only the cells treated with erastin presented a very low level of intracellular glutathione which persisted even 24 h after washing out the inhibitor (Fig. 3B).

**Figure 4:**
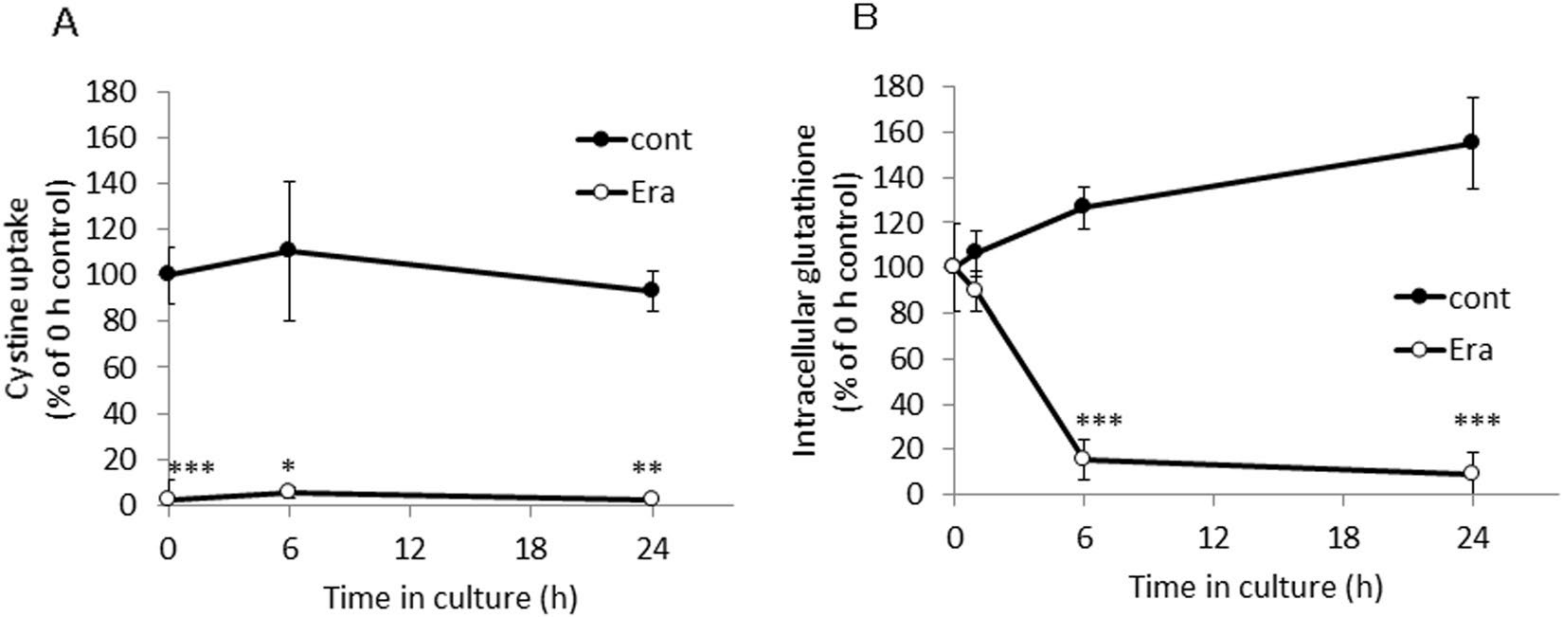
Time course of the change of the activity of cystine uptake (**A**) and intracellular glutathione (**B**) in xCT-overexpressing MEF exposed to erastin for a short time. xCT-overexpressing MEF were cultured for 24 h and subsequently exposed to 100 µM erastin for 5 min. After exposure, cells were washed with 1 mL fresh medium once. Two mL of fresh medium was added (regarded as 0 h) and cells were allowed to grow for 24 h. At the time periods indicated, the uptake of 0.05 mM L-[^14^C]cystine (**A**) and/or intracellular glutathione levels (**B**) were measured. Each point represents the mean of percentages ± S.D. (n = 6 for 0 h control; n = 4 for other points). P values were obtained by unpaired Student’s t test. ^*^P = 1.5 × 10^−2^ (control vs erastin at 6 h of cystine uptake), ^**^P = 3 × 10^−3^ (control vs erastin at 24 h of cystine uptake), ^***^P = 1 × 10^−7^ (control vs erastin at 0 h of cystine uptake determination), 5 × 10^−5^ (control vs erastin at 6 h of intracellular glutathione determination), 1 × 10^−4^ (control vs erastin at 24 h of intracellular glutathione determination).

Next, we performed time course experiments to further explore the mechanism of inhibition and its impact on cellular glutathione levels. As illustrated in Fig. 4A, a 5 min exposition of cells to erastin sufficed to obtain a strong inhibitory effect towards xCT which persisted over the entire observation time. This strong and persistent inhibitory effect was followed by a massive drop of intracellular glutathione concentrations which reached its lowest levels as early as 6 h upon erastin treatment (Fig. 4B). It was, however, not possible to explore if the inhibitory effect of erastin would have lasted for longer time periods as the cells started to die after 24 h under the routine cell culture conditions due to strongly decreased intracellular glutathione concentrations.

### Sensitivity to erastin by sulfhydryl modification of each cysteine residue of xCT

**Figure 5:**
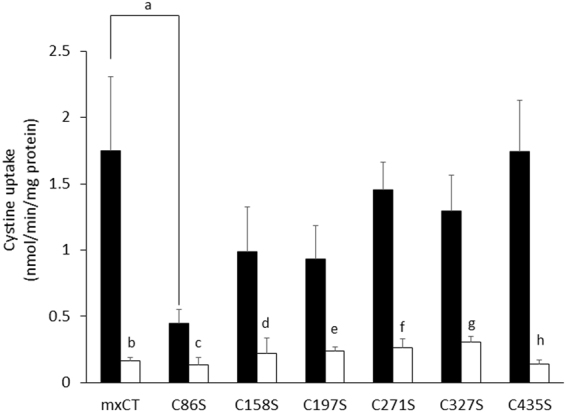
Effects of erastin on the activity of cystine uptake in xCT-deficient MEF expressing xCT with different Cys to Ser substitutions. xCT-deficient MEF were cultured for 24 h and then plasmids containing either wild-type xCT or the different Cys to Ser mutants in pEF-BOS were transfected into cells. After 48 h incubation, the uptake of 0.05 mM L-[^14^C]cystine was measured in the absence (filled bar) or presence (open bar) of 10 µM erastin. Bars represent the mean ± S.D. (n = 8 for wild-type xCT (mxCT); n = 7 for other mutant plasmids). P values were obtained by unpaired Student’s t test. Each small letters indicate statistical significance between mxCT and C86S in the absence of erastin, between the absence and presence of erastin in each cells. P values are a = 5 × 10^−4^, b = 2 × 10^−4^, c = 2 × 10^5^, d = 1 × 10^−3^, e = 4 × 10^−4^, f = 1 × 10^−8^, g = 6 × 10^−6^, h = 8 × 10^−6^.

The results obtained above suggested that erastin irreversibly binds to xCT protein. If this is the case, it might be that erastin covalently reacts with sulfhydryl group of cysteine residues. To this end, xCT mutant forms were engineered by replacing the individual six endogenous cysteine residues with serine as described in Materials and Methods. All six mutant forms of xCT were expressed in xCT-deficient MEF and no differences of proliferation rates were detectable between the different cell lines (not shown). As shown in Fig. 5, xCT-deficient MEF transfected with each cysteine-mutant showed somewhat different activity of cystine uptake in the absence of erastin. However, the cystine uptake activity in all of these cell lines could be equally and potently inhibited by erastin, indicating that erastin does not inhibit cystine uptake by reacting with a single sulfhydryl group of any of these cysteine residues.

### Effect of erastin on proliferation of cisplatin-resistant human ovarian cancer cells

**Figure 6:**
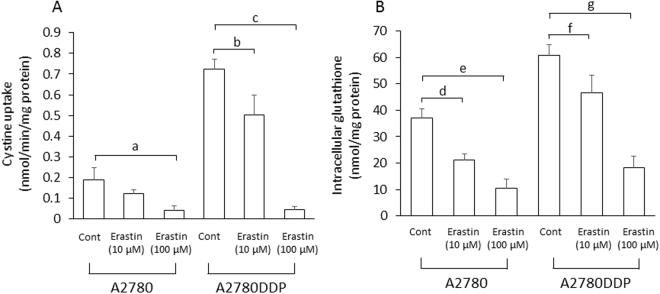
Effect of a short time pre-exposure of erastin on the activity of cystine uptake and intracellular total glutathione in human ovary cell lines. A2780 and A2780DDP cells were seeded on 35 mm dishes (2 × 10^5^ cells/dish), cultured for 24 h and exposed to 10 µM or 100 µM erastin for 5 min. After removing erastin from the medium, the cells were washed with 1 mL of fresh medium once and were allowed to grow in fresh medium in the presence or absence of 10 µM cisplatin. After culturing the cells for 24 h, the uptake of 0.05 mM L-[^14^C]cystine (**A**) and intracellular glutathione levels (**B**) were measured. Each point represents the mean ± S.D. (n = 4). P values were obtained by unpaired Student’s t test. Each small letters indicate statistical significance. P values are a = 6 × 10^−3^, b = 3.6 × 10^−2^, c = 1 × 10^−4^, d = 6 × 10^−3^, e = 2 × 10^−3^, f = 1 × 10^−3^, g = 3 × 10^−4^.

**Figure 7:**
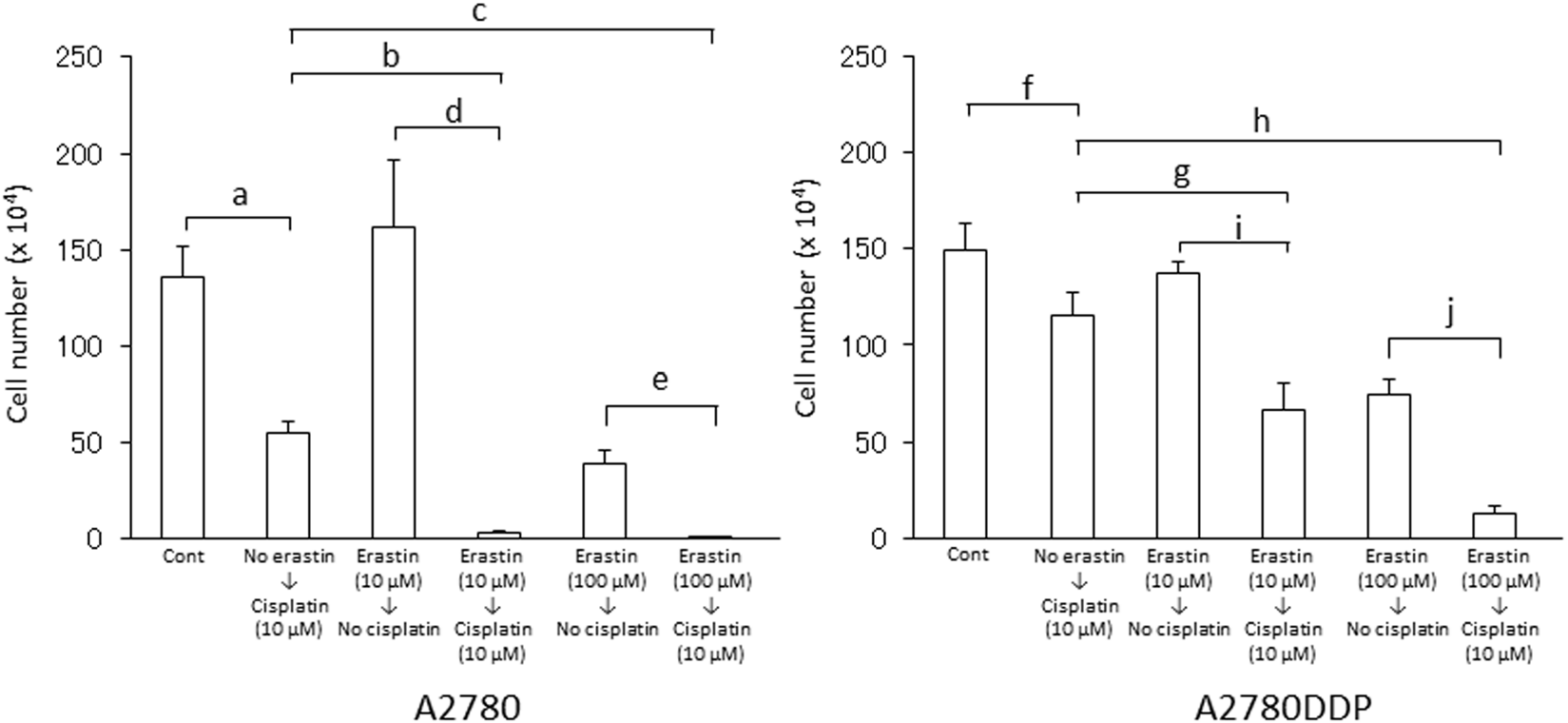
Effect of a short time pre-exposure of erastin on proliferation of human ovary cell lines in the presence of cisplatin. A2780 (left panel) and A2780DDP (right panel) cells were seeded on 35 mm dishes (2 × 10^5^ cells/dish), cultured for 24 h, and exposed to 10 µM or 100 µM erastin for 5 min. After removing erastin from the medium, the cells were washed with 1 mL of fresh medium once and were allowed to grow in fresh medium in the presence or absence of 10 µM cisplatin. After culturing the cells for 48 h, the number of viable cells was counted by the trypan blue-exclusion method. Bars represent the mean of cell number ± S.D. (n = 10 for control of A2780 and A2780DDP; n = 4 for other conditions). P values were obtained by unpaired Student’s t test. Each small letters indicate statistical significance. P values are a = 2 × 10^−3^, b = 3 × 10^−4^, c = 4 × 10^−4^, d = 3 × 10^−3^, e = 2 × 10^−3^, f = 3 × 10^−3^, g = 1.5 × 10^−3^, h = 9 × 10^−4^, i = 2 × 10^−3^, j = 2 × 10^−4^.

We previously investigated the activity of cystine uptake and intracellular glutathione in human ovarian cell line (A2780) and its cisplatin (CDDP)-resistant counterpart (A2780DDP), and found that the activity of cystine uptake and intracellular glutathione in A2780DDP cells were significantly higher than those in A2780 cells^22^. Using both cell lines, we investigated the pre-treatment effect of erastin. After 5 min incubation with 10 or 100 μM erastin, the cell culture medium was removed, cells were rinsed once with 1 ml of fresh medium and incubated with 2 ml of fresh medium lacking any inhibitors for 24 h. After 24 h, the cystine uptake was measured in the absence of erastin. At the same time, intracellular glutathione levels were determined. As shown in Fig. 6A, the uptake of cystine was still inhibited in a dose-dependent manner in both types of cells which had been exposed for just 5 min with erastin. Accordingly, the cells treated with erastin presented a low level of intracellular glutathione which persisted even 24 h after washing out the inhibitor (Fig. 6B). Then, we performed a side by side comparison study and explored the sensitivity of cisplatin in these cells which had been exposed to erastin for a short time (Fig. 7). When A2780 cells were cultured for 48 h with 10 μM cisplatin alone, the cell number was decreased to approximately 36% of control cells (no additives), whereas A2780DDP cells showed significant resistance to 10 μM cisplatin, and the cell number was decreased to only approximately 73% of control cells. When the cells were exposed to 10 μM erastin for 5 min and the cells were allowed to grow for another 48 h after the short erastin treatment, cell numbers of A2780 and A2780DDP cells were approximately 82% and 74% of cell number of untreated control cells, respectively. On the other hand, when the cells were exposed to 10 μM erastin for 5 min, and allowed to grow for another 48 h in the presence of 10 μM cisplatin alone after removing erastin by washing out from the medium, cell numbers of A2780 and A2780DDP cells were reduced to approximately 2% and 42% of the cell number of control cells, respectively. These results indicate that most of A2780 cells died and the survival of A2780DDP cells was significantly suppressed under these conditions. When the cells were exposed to 100 μM erastin for 5 min and allowed to grow for another 48 h in the presence of 10 μM cisplatin alone after removing erastin, the sensitivity of the cells to cisplatin was drastically increased. Under these conditions, cell numbers of A2780 and A2780DDP cells were only 0.02% and 8% of the cell number of control cells. These results show that A2780 cells almost completely died and only a minority of A2780DDP cells survived this combination treatment. Taken together, the present results indicate that the cell death inducing effects of cisplatin can be strongly potentiated by a short bolus treatment of cells with erastin.

## Discussion

Erastin was first reported as a small molecule compound efficiently killing human tumor cells without killing their isogenic normal cell counterparts^19^. Dixon and colleagues further found that erastin causes a novel iron-dependent form of regulated necrotic cell death, designated as ferroptosis^20^. They also showed that erastin is a potent and selective inhibitor of system x_c_^−^^21^. In addition to their data that erastin has no impact on system L-mediated phenylalanine uptake, our present study now shows that erastin does not affect system ASC-mediated serine uptake and system y^+^-mediated arginine uptake (Fig. 1). We further demonstrate that a short time exposure of erastin is sufficient to confer the inhibitory effect of erastin towards cystine uptake via system x_c_^−^ inhibition, and consequently deprivation of intracellular glutathione (Fig. 3). Cystine uptake via xCT is competitively blocked by substrates of system x_c_^−^, such as L-glutamate, α-aminoadipate, L-homocysteate, and L-cystathionine^8^. In addition to these substrates, several non-substrate inhibitors, such as (S)-4-carboxyphenylglycine were described by Patel and colleagues^23^. They also analyzed analogues of amino methylisoxazole propionic acid as the inhibitor of system x_c_^−^^24^. On the other hand, Gout, *et al*., found that the anti-inflammatory drug sulfasalazine is a potent and specific inhibitor of this transport system^25^. Compared to these known inhibitors, erastin inhibits cystine uptake much more efficiently with an IC_50_ value of around 1.4 µM (Fig. 2). Moreover, we provide strong evidence that the inhibitory effect of erastin on cystine uptake via system x_c_^−^ lasts at least for 24 h after removing erastin from the cell culture medium. Probably due to the continuous inhibition against cystine uptake, intracellular glutathione concentrations were found to be sharply decreased and remained at very low levels for 24 h under these conditions (Fig. 4). No other known xCT inhibitor has ever shown such characteristics. These results strongly suggest that erastin irreversibly binds to and thereby inactivates xCT.

Jiménez-Vidal, *et al*., performed cysteine scanning accessibility studies to unravel the mechanism of the inhibitory effects of mercurial reagents, such as pCMB and pCMBS, which also potently inhibit system x_c_^−^ activity. Their results indicated that these mercurial reagents react with Cys327 of human xCT, because an xCT mutant with a cysteine to serine substitution at Cys327 had a similar activity of glutamate uptake as wild-type xCT in the presence of pCMB or pCMBS^26^. Therefore, we hypothesized that if erastin reacts with a certain part of the xCT protein in a manner like the mercurial reagents, it might be that the chlorobenzene part of erastin may be replaced with sulfhydryl groups of certain cysteine residues of xCT. Yet, as shown in Fig. 5, a strong inhibitory effect of erastin was observed in the xCT-deficient cells regardless which mutant form of xCT they expressed. These findings suggest that erastin does not react with any of the sulfhydryl group of cysteine residue in the xCT protein, although the possibility that erastin reacts with more than one cysteine residue to inhibit cystine uptake cannot be formally ruled out. Yagoda, *et al*., previously showed that erastin acts by binding directly to mitochondrial voltage-dependent anion channels (VDACs)^27^. Thus, this compound seems to have the property of binding membrane proteins somehow, although the molecular mechanism of how erastin interacts with xCT needs further investigations. Interestingly, when the C86S construct is expressed in the cells, the activity of cystine uptake was significantly decreased, as compared with the wild-type construct. Cys86 is located in the middle part of transmembrane domain 2 by topology predictions^26^, and a similar tendency was observed in the case of human xCT^26^. The sulfhydryl group of this cysteine residue may thus be involved in the permeation pathway of the substrates.

Recent studies have suggested that xCT is involved in cancer pathology^13^. Gout, *et al*., showed that inhibiting cystine uptake via system x_c_^−^ arrests proliferation of lymphoma cell lines *in vitro*^28^. In many human cancer cell lines, high expression of xCT is observed^29^. Chung, *et al*., demonstrated that sulfasalazine inhibits the growth of transplanted glioma cells in mice^30^, and that glutamate released via system x_c_^−^ acts as an autocrine and/or paracrine signal for promoting glioma cell invasion^31^. Savaskan, *et al*., also reported that xCT confers a crucial role in glioma-induced neurodegeneration and brain edema^32^. On the other hand, xCT is stabilized by interacting with the splice variant of CD44, one of surface maker molecules associated with cancer stem cells, causing enhanced glutathione level and an increased defense against reactive oxygen species in human gastrointestinal cancer cells^33^. Recently, the major tumor suppressor gene, p53, has been reported to inhibit cystine uptake and sensitize cells to ferroptosis by repressing expression of xCT in human cancer cell lines^34^. Therefore, potent and highly specific inhibitors of xCT have been regarded as useful drugs for cancer chemotherapy. From this point of view, a variety of compounds have been investigated, including sulfasalazine analogs^35^. However, the clinical trial of sulfasalazine for patients with gliomas was unsuccessful due to the lack of clinical response and severe side effects^36,37^. Recently, Roh, *et al*., have demonstrated that inhibition of xCT function enhances cisplatin cytotoxicity of cisplatin-resistant head and neck cancer cells^38^. Their results are consistent with those of our present study. Here, we demonstrate that a brief erastin exposure of tumor cells is sufficient to dramatically enhance cisplatin’s cytotoxicity in cisplatin-resistant ovary cancer cells. It is likely that a compound like erastin, which persistently blocks cystine uptake, might show beneficial and synergistic effects in cancer treatment regimens by applying it just before the onset of other anticancer drugs. Dixon, *et al*., have performed a structure activity relationship analysis of erastin and described some improved analogs with increased inhibitory effects towards system x_c_^−^^21^. In case these compounds confer the same persistent inhibitory effect on xCT similarly like erastin, they may be expected to be used at lower concentrations before treating patients with anticancer drugs to increase chemotherapy efficacy.

## Methods

### Materials

Erastin was obtained from Sigma (St. Louis, MO, U.S.A.), sulfasalazine from LKT Laboratories, Inc (St. Paul, MN, U.S.A.) and (S)-4-carboxyphenylglycine from Tocris Bioscience (Birmingham, UK). Quisqualic acid was obtained from Sigma (St. Louis, MO, USA). All other chemicals and regents were purchased from Wako Pure Chemical Industries, Ltd. (Osaka, Japan), unless stated otherwise.

### Cell culture

xCT-overexpressing mouse embryonic fibroblasts (MEF) were generated by stable transfection of cells with pCAG-3SIP-based vector containing the coding region of murine xCT^11,39^ in a manner similarly as described by Lewerenz, *et al*.^40^. pCAG-3SIP-based vector contain a strong hybrid promoter, consisting of the chicken β-actin promoter and the CMV enhancer, which is highly active in many cell types including fibroblasts^41^. xCT-overexpressing MEF and xCT-deficient MEF^42^ were cultured routinely in Dulbecco’s modified Eagle’s medium supplemented with 10% fetal bovine serum, penicillin (50 U/mL) and streptomycin (50 µg/mL) at 37 °C in 5% CO_2_ and 95% air. xCT-overexpressing MEF were seeded on 35 mm dishes (2 × 10^5^ cells/dish), cultured for 24 h, and then measured for amino acid uptake activities in the presence or absence of xCT inhibitors at the concentrations indicated. When the cells were exposed to xCT inhibitors for a short time (5 min, 24 h after seeding xCT-overexpressing cells on the dishes), the medium was removed, and the cells were washed with fresh medium once, and then 2 ml of fresh medium was added. After culturing the cells at the time periods indicated, the activity of cystine uptake was measured in the absence of inhibitors. Under the same conditions, intracellular glutathione was measured.

Human ovarian cancer cell line (A2780 and its CDDP-resistant variant A2780DDP) were donated by Dr. K J Scanlon (Biochemical pharmacology, City of Hope National Medical Center, Duarte, CA, U. S. A.). These cells were cultured in RPMI1640 supplemented with 5% fetal bovine serum at 37 °C in 5% CO_2_. The CDDP-resistant cells were treated with 100 µM CDDP for 2 h every week as described^43^.

### Measurement of amino acid transport activity

Cells were washed three times in pre-warmed PBS(+)G (10 mM phosphate-buffered saline pH 7.4, containing 0.01% CaCl_2_, 0.01% MgCl_2_ · 6H_2_O and 0.1% glucose) and then incubated in 0.5 ml of pre-warmed uptake medium at 37 °C for 2 min. The uptake medium contained 50 μM cystine plus [^14^C]cystine (0.2 μCi/mL), 50 µM leucine plus [^14^C]leucine (0.2 µCi/mL), 50 µM serine plus [^14^C]serine (0.2 µCi/mL) or 50 µM arginine plus [^14^C]arginine (0.2 µCi/mL) in PBS(+)G. Erastin, sulfasalazine, (S)-4-carboxyphenylglycine, quisqualic acid or L-glutamate each at final concentrations indicated were added to the uptake medium as inhibitors. Uptake was terminated by rapidly rinsing the cells three times with ice-cold PBS, and the radioactivity in the cells was counted by the liquid scintillation counter (LSC-5100, ALOKA, Japan).

### Determination of intracellular total glutathione (GSH and the oxidized form of GSH (GSSG))

Cells were rinsed three times in ice-cold phosphate-buffered saline (pH 7.4). Intracellular glutathione was extracted with 5% trichloroacetic acid and then treated with ether to remove the acid. Total glutathione content in the aqueous layer was measured using an enzymatic method described previously, which is based on the catalytic action of GSH in the reduction of 5,5′-dithiobis(2-nitrobenzoic acid) by the GSH reductase system^44^.

### Site-directed mutagenesis

Mouse xCT cDNA cloned in pEF-BOS^45^ was used as template. Six endogenous cysteine residues of mouse xCT were individually mutated to serine using PrimeSTAR^®^ Mutagenesis Basal Kit (TaKaRa Bio, Inc, Shiga, Japan) according to the manufacturer’s instructions. pEF-BOS was kindly donated by Kazuichi Sakamoto (University of Tsukuba, Ibaraki, Japan). Primer sets used for the site-directed mutagenesis studies are shown in Table 1.

**Table 1:** Primer sets using for site-directed mutagenesis.

C86S	forward	5′-TCTGCCTCTGAAGTACTGTCACTTTTT-3′
	reverse	5′-TACTCCAGAGGCAGACCAGAAAACCAG-3′
C158S	forward	5′-ATTCAATCTGAAATTCCTGAACTTGCA-3′
	reverse	5′-AATTTCAGATTGAATAAAAAATGGTTCC-3′
C197S	forward	5′-ACCTTTTCCAAGCTCACAGCAATTCTG-3′
	reverse	5′-GAGCTTGGAAAAGGTTAGGAAAATCTG-3′
C271S	forward	5′-GCAATCTCCATCTCCATGGCTATCATC-3′
	reverse	5′-GGAGATGGAGATTGCAAGGGGGATGGT-3′
C327S	forward	5′-CTCTCCTCCTTCGGCTCCATGAACGGT-3′
	reverse	5′-GCCGAAGGAGGAGAGGGCAACAAAGAT-3′
C435S	forward	5′-TTCACCTCCCTCTTCATGGTCGTCCTC-3′
	reverse	5′-GAAGAGGGAGGTGAAGGAAAATAGTGC-3′

### Transfection

After seeding 1.0 × 10^5^ cells/35-mm dish and culturing for 24 h, xCT-deficient cells were transfected with 5 µg of each mutated xCT plasmid using Lipofectamine® 3000 Transfection Reagent (Life Technologies, Carlsbad, USA) according to the manufacturer’s instructions. After 48 h incubation, the activity of cystine uptake was measured as mentioned above.

### Determination of cell numbers

Cell viability was measured by trypan blue staining. After washing cells with PBS three times, cells were trypsinized and re-suspended in 1 ml of fresh medium. Then, an equal volume of 0.1% trypan blue solution was added to 50 µL of the cell suspension and viable cell numbers were counted with a hemocytometer.

### Statistical analyses

Statistical significances of the differences were determined by Student’s t test. For multiple comparison procedures, one-way ANOVA followed by Tukey’s multiple comparison test was used.

## Electronic supplementary material

Supplementary information
