# Supplementary material for: The ferroptosis inducer erastin irreversibly inhibits system xc− and synergizes with cisplatin to increase cisplatin’s cytotoxicity in cancer cells
Source: Sci Rep. 2018 Jan 17;8:968. doi: 10.1038/s41598-018-19213-4 (PMC5772355; doi:10.1038/s41598-018-19213-4)
Supplement: Supplementary file 1 — Supplementary information [file 41598_2018_19213_MOESM1_ESM.pdf]

The ferroptosis inducer erastin irreversibly inhibits system x_c^- and synergizes with cisplatin to increase cisplatin's cytotoxicity in cancer cells

Mami Sato[1, 3], Ryosuke Kusumi[1], Shinji Hamashima[2, 3], Sho Kobayashi[2, 3], Satoru Sasaki[3], Yuhei Komiyama[3], Takuji Izumikawa[4], Marcus Conrad[5], Shiro Bannai[1], and Hideyo Sato[1*]

Supplementary Information

Supplementary Fig. S1. Effect of erastin on the activity of cystine uptake in xCT-over-expressing and wild-type MEF. xCT-overexpressing (OE) and wild-type (WT) MEF were cultured for 24 h and subsequently incubated in the absence or presence of 100 μM erastin for 5 min. After the incubation with erastin, the uptake of 0.05 mM L-[^{14}C]cystine was measured in the absence of erastin. The percent inhibition of the uptake of cystine in OE-MEF and WT-MEF treated with erastin was 97.9% and 75.2%, respectively. Data represent the mean of the activity of cystine uptake ± S.D. in OE-MEF and the mean of the activity of cystine uptake in WT-MEF. Each point represents the mean ± S.D. (n=4). P values were obtained by unpaired Student's t test. ***P=5 x 10^{-4} (OE-MEF vs OE-MEF + Erastin), 2 x 10^{-4} (WT-MEF vs WT-MEF + Erastin).

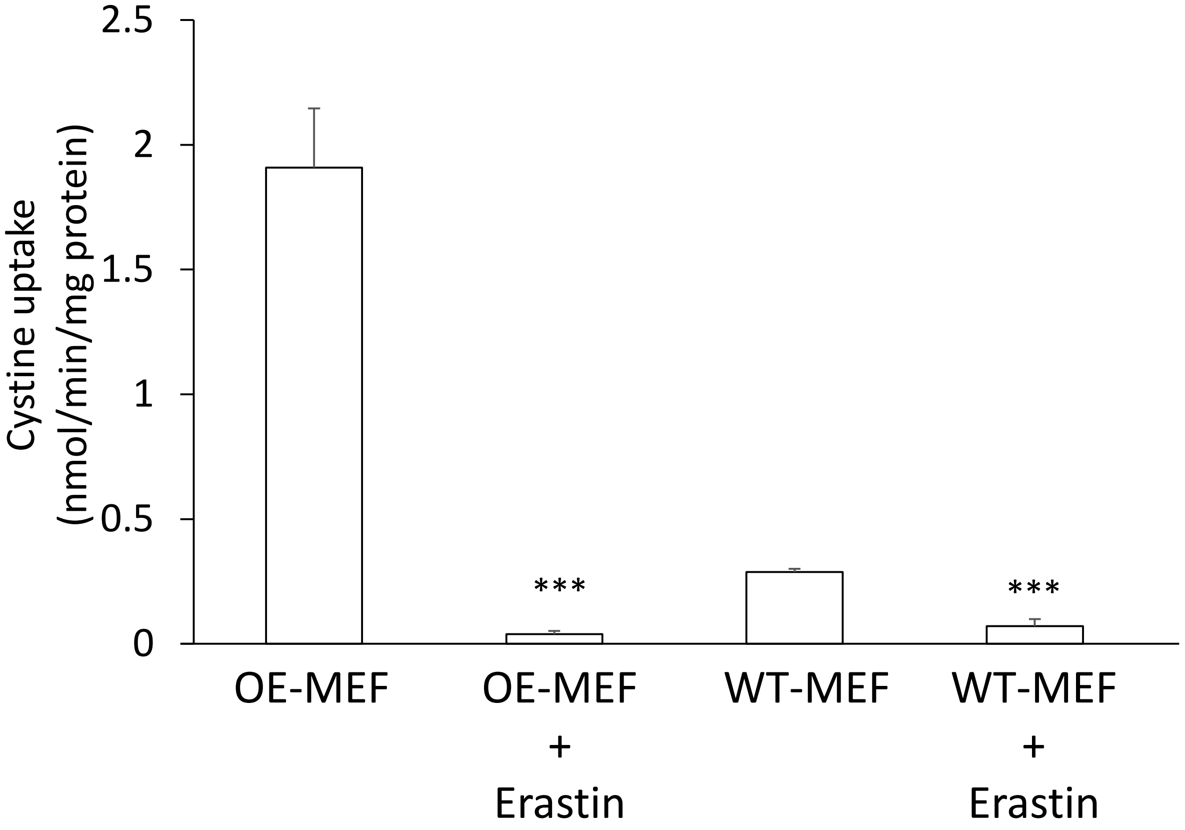

Supplementary Fig. S1
